# Supplementary figures and images for: Multiplexed assays of human disease-relevant mutations reveal UTR dinucleotide composition as a major determinant of RNA stability
Source: eLife. 2025 Feb 18;13:RP97682. doi: 10.7554/eLife.97682 (PMC11835390; doi:10.7554/eLife.97682)

# Post-transfection

M

1<sup>st</sup> 2<sup>nd</sup> 3<sup>rd</sup> time points

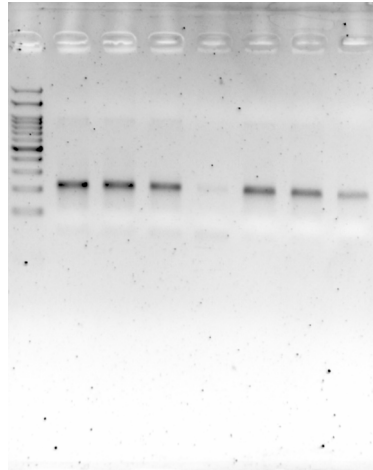

Supplement: Figure 1—source data 1. [file elife-97682-fig1-data1.zip › Figure 1_source data 1/Figure 1_source data 1.pdf]

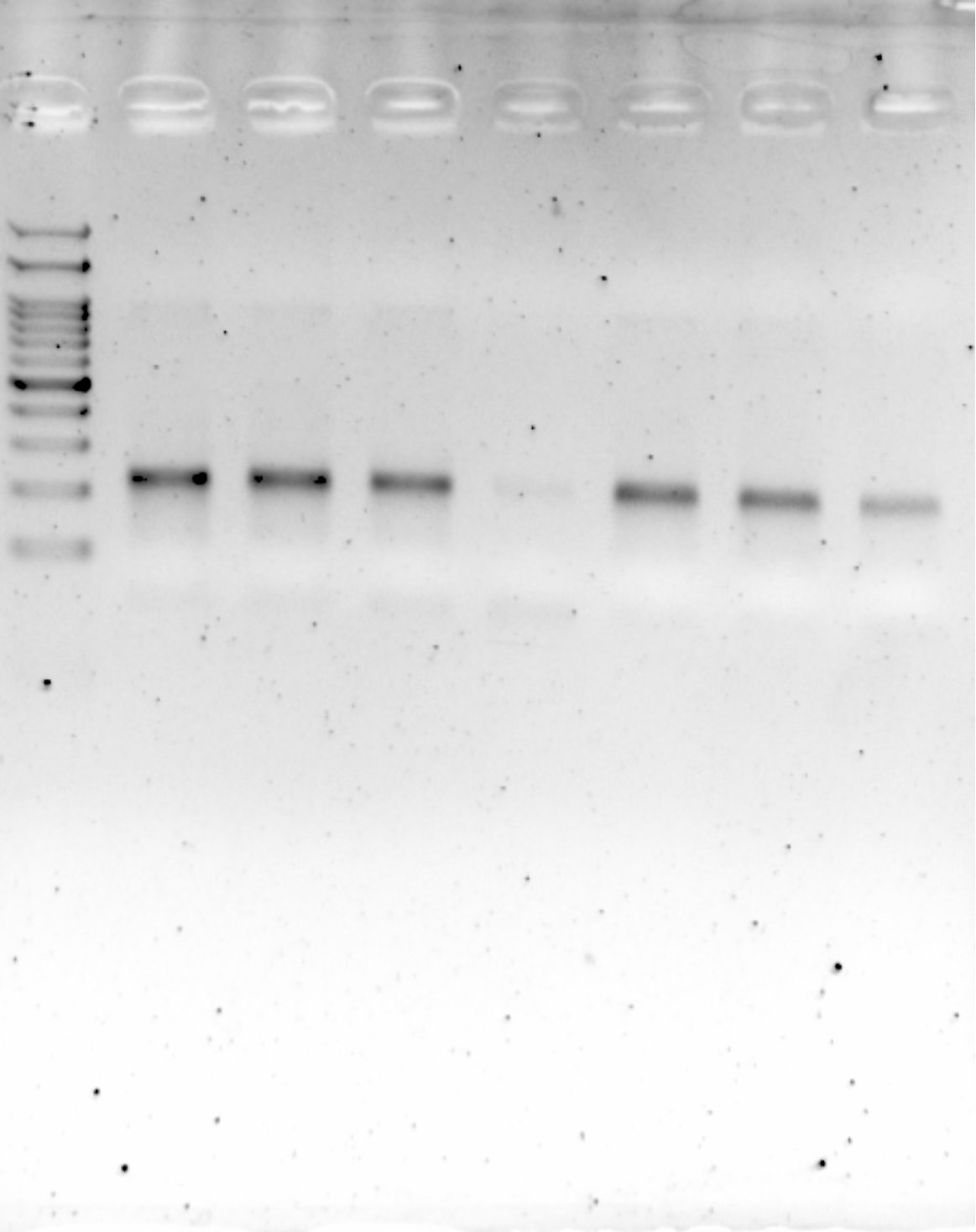

Supplement: Figure 1—source data 2. [file elife-97682-fig1-data2.zip › Figure 1_source data 2/CLL 2018-11-11_16h31m33s_5'UTR_2nd_short_828_30min_9.1_9.14_40X.tif]

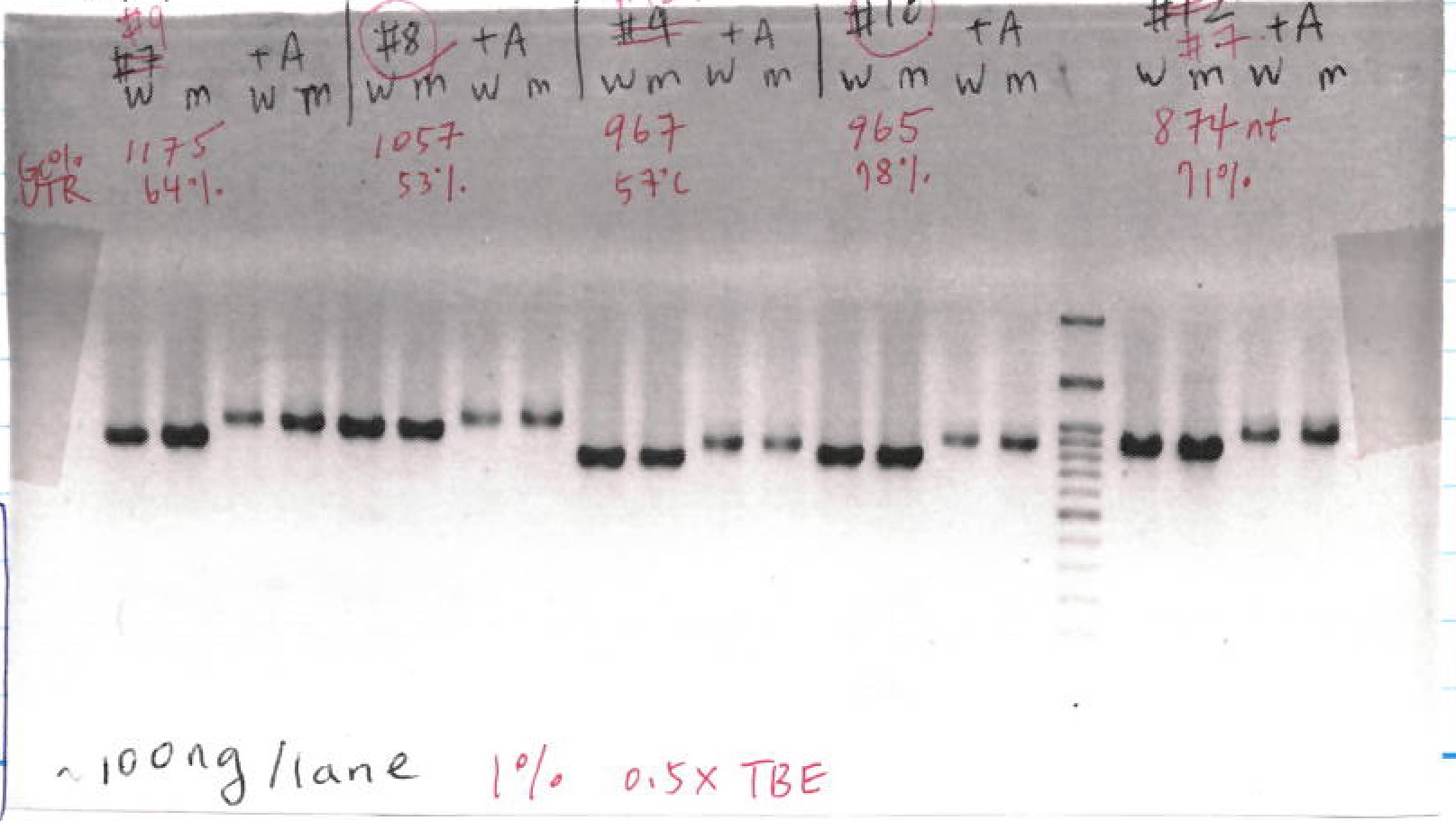

Supplement: Figure 1—figure supplement 1—source data 1. [file elife-97682-fig1-figsupp1-data1.zip › Figure 1-figure supplment 1-source data 1/Figure 1-figure supplement 1C.jpg]

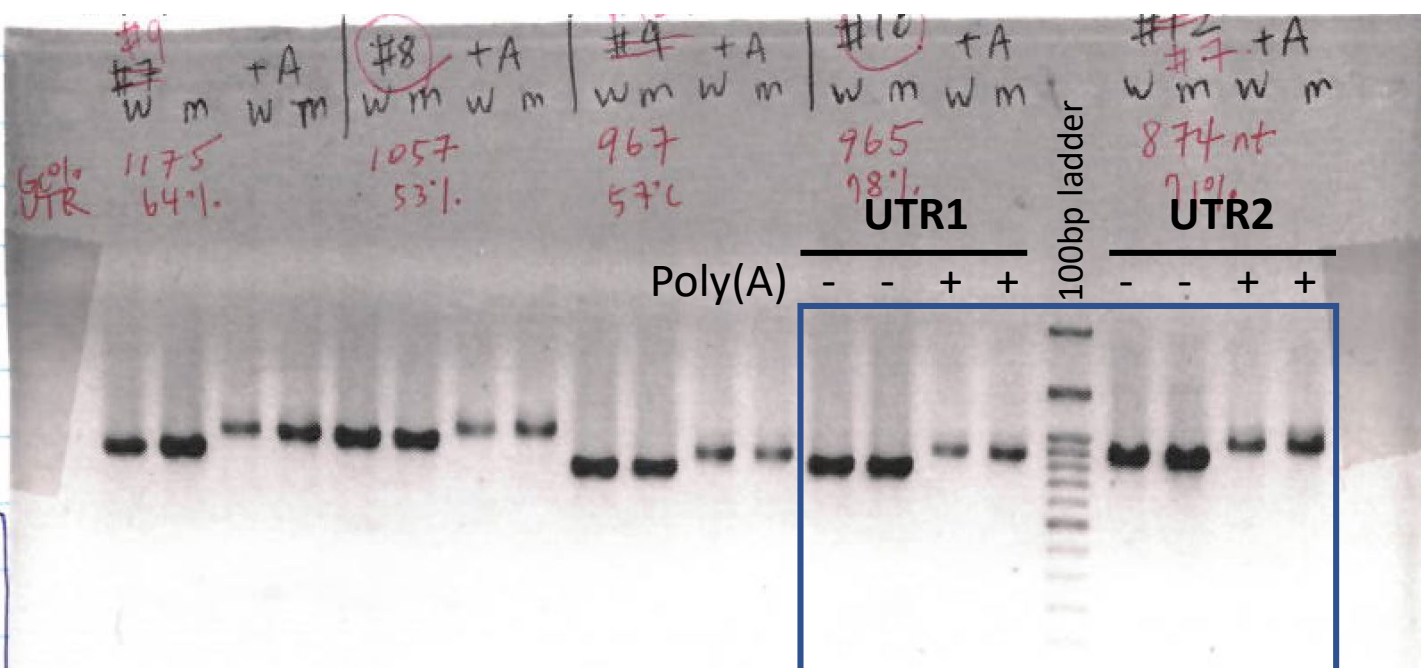

~100ng/lane 1% 0.5x TBE

Supplement: Figure 1—figure supplement 1—source data 2. [file elife-97682-fig1-figsupp1-data2.zip › Figure 1-figure supplment 1-source data 2/Figure 1-figure supplment 1-source data 1-labeled.pdf]
